# Feasibility of Unilateral Approach for Bilateral Decompressive Endoscopic Spinal Surgery for Lumbar Stenosis to Improve Back and Leg Pain: A Consecutive Single-Center Series of 60 Patients

**DOI:** 10.3389/fsurg.2020.507954

**Published:** 2020-12-08

**Authors:** Azizul Akram Salim, Abdul Halim Yusof, Joehaimey Johari, Mohd Imran Yusof

**Affiliations:** ^1^Hospital Universiti Sains Malaysia, Universiti Sains Malaysia, Kota Bharu, Malaysia; ^2^Department of Orthopaedics, School of Medical Sciences, Universiti Sains Malaysia, Kubang Kerian, Malaysia

**Keywords:** neurological outcome, lumbar stenosis and radicular pain, endoscopic decompression, minimally invasive surgery (MIS), endoscopic spine surgery

## Abstract

**Introduction:** Endoscopic surgery is one of the methods that achieve the goal of decompression while minimizing collateral tissue damage. Its efficacy and safety have been supported by numerous studies. There is a plethora of studies on lumbar stenosis regarding the outcomes and related issues in endoscopic spine surgery. However, few studies evaluated the outcome of the decompressive lumbar spine surgery. The present study aims to analyze the outcome of a unilateral approach to endoscopic surgery for lumbar stenosis using the visual analog scale (VAS), the Oswestry Disability Index (ODI), and MacNab's criteria.

**Methods:** This is a retrospective study (level IV) conducted between January 2009 and December 2013 on 60 patients who underwent endoscopic interlaminar decompressive spine surgery (Destandau method) for lumbar degenerative spinal stenosis in the Hospital Universiti Sains Malaysia. The clinical outcome was measured pre-operatively and post-operatively for VAS: for back and leg pain, motor and sensory grading, the ODI, and MacNab's criteria. A paired *t*-test was used for statistical analysis.

**Results:** The mean age of patients was 60.82 years comprising 23 males (38.3%) and 37 females (61.7%). The mean follow-up period was 30.1 months (range = 17.2–43 months). The mean operation time was 183.6 min (ranging from 124.8 to 242.4 min), and the mean blood loss was 150.18 mL (ranging from 30.82 to 269.54 mL). Post-operatively, mean hospital stay was 2.45 days (ranging from 1.34 to 3.56 days). The most frequently involved level was L4/L5 in 51 patients (52.6%), followed by L3/L4 in 19 patients (19.6%), L5/S1 in 24 patients (24.7%), and L2/L3 in three patients (3.1%). Improvement in the post-operative VAS for back and leg pain and the ODI for pre-operation and post-operation was statistically significant (*p* < 0.001). Conversely, the reduction in neurological status was statistically insignificant. Based on MacNab's criteria, 88.4% showed excellent to good outcomes.

**Conclusion:** To summarize, unilateral percutaneous endoscopic spine surgery to achieve the bilateral decompression in lumbar stenosis provides excellent yet safe and effective outcomes. It improves back and leg pain and patients' function significantly.

## Introduction

Lumbar spinal stenosis (LSS) is a disease pathology that emerges from various sites such as the intervertebral disc, capsule, bone, and ligament. A combination of the mentioned factors can cause a compression to the lumbar spinal canal, which results in the clinical symptoms of neurogenic claudication and radiculopathy and eventually leads to cauda equina syndrome. Various hypotheses intended to explain the onset of the pain: nerve, vascular, inflammatory, and biochemical components (1–3).

For decades, decompressive surgery has been the gold standard and common treatment for lumbar stenosis. However, endoscopic surgery is becoming prominent in the decompressive surgery arena. The evolution of minimally invasive lumbar decompression has started since the 1960s. Despite the conventional open decompression, Kambin and Gellman (4) had used the posterior transcanal endoscopic by using Craig cannula in 1973. A few years later, in 1975, Hijikata et al. (5) introduced a standalone procedure of a non-visualized posterolateral percutaneous nucleotomy followed by Kambin and Gellman (4), who reported nine cases of similar procedure in 1983. Then, in 1985, Onik et al. (6) conducted nucleotomy by using a 2.8-mm-diameter shaver. Later in 1989, Schreiber et al. (7) injected a dye (indigo carmine) to blue stain the pathological nucleus pulposus and annular fissure. In 1998, Kambin et al. (8) used the transforaminal biportal approach to excise central herniation and non-migrated sequestrated disk fragments in 59 cases.

As for the tissue damage, a comparative one-on-one study by Shin compared groups of microendoscopic discectomy (MED) and standard microscopic discectomy (MD) (9). Post-operatively, the creatine phosphokinase–MM isoenzyme mean level was lower in the MED group than in the MD group at 3 and 5 days (*p* < 0.05), respectively. Similarly, the visual analog scale (VAS) score for post-operative back pain was lower than MED in both 1 and 5 days (*p* < 0.01). The present study concluded that MED caused less muscle damage and backache.

However, the outcomes of the abovementioned surgeries reported in the Western population were poorly documented. Presently, some studies in the Korean context discussed endoscopic lumbar surgery, but with focusing on endoscopic lumbar discectomy surgery. Thus, the present study intends to fill in the gap of the minimal literature on the clinical outcomes of endoscopic lumbar stenosis surgery, specifically in the Asian population. The present study also aims to determine the clinical outcomes of endoscopic surgery by using the unilateral hemilaminotomy and bilateral decompression approach for LSS. The aims of the present study can be achieved by determining the improvement of pain using the VAS, motor and sensory function, MacNab's criteria, and the Oswestry Disability Index (ODI) pre-surgery and post-surgery.

## Materials and Methods

This retrospective study was conducted between January 2009 until December 2013 at the Hospital Universiti Sains Malaysia and was approved by the Human Research Ethics Committee of USM (JEPeM) (JEPeM code: USM/JEPeM/14070258). Sixty patients 45 years or older were involved in the present study as one cohort. The patients were diagnosed with single or multiple levels of stenosis, clinical symptoms, and radiological findings on magnetic resonance imaging for lumbar stenosis and underwent interlaminar endoscopic surgery (Destandau technique). The cohort was followed up for at least 1 year after their respective surgeries. Patients with previous open decompression surgery; underlying pain-generated diseases, for example, rheumatoid arthritis, and fibromyalgia; and significant spine instability were excluded. The sample size was calculated using Power and Sample Size Calculations Software Version 3.0, with options for comparison of two dependent means. Ahn et al. (10) reported that the standard deviation of difference for using VAS as the outcome of interest was 1.55. For a difference of 0.93 unit for VAS between pre-measurement and post-measurement to be statistically significant (effect size = 0.6) at 5% probability of type I error and 20% probability of type II error (80% power of study), the required calculated sample size was 48 patients. Anticipating a 20% dropout rate due to missing data, the corrected sample size was 58 patients. A paired-sample *t*-test was then used for statistical analysis to compare differences between pre– and post–endoscopic surgery in terms of low back and leg pain. Once the assumptions for normality of the differences were checked and fulfilled, a paired-sample *t*-test was then conducted. There was no adjustment of confounders being conducted.

**Figure 1:**
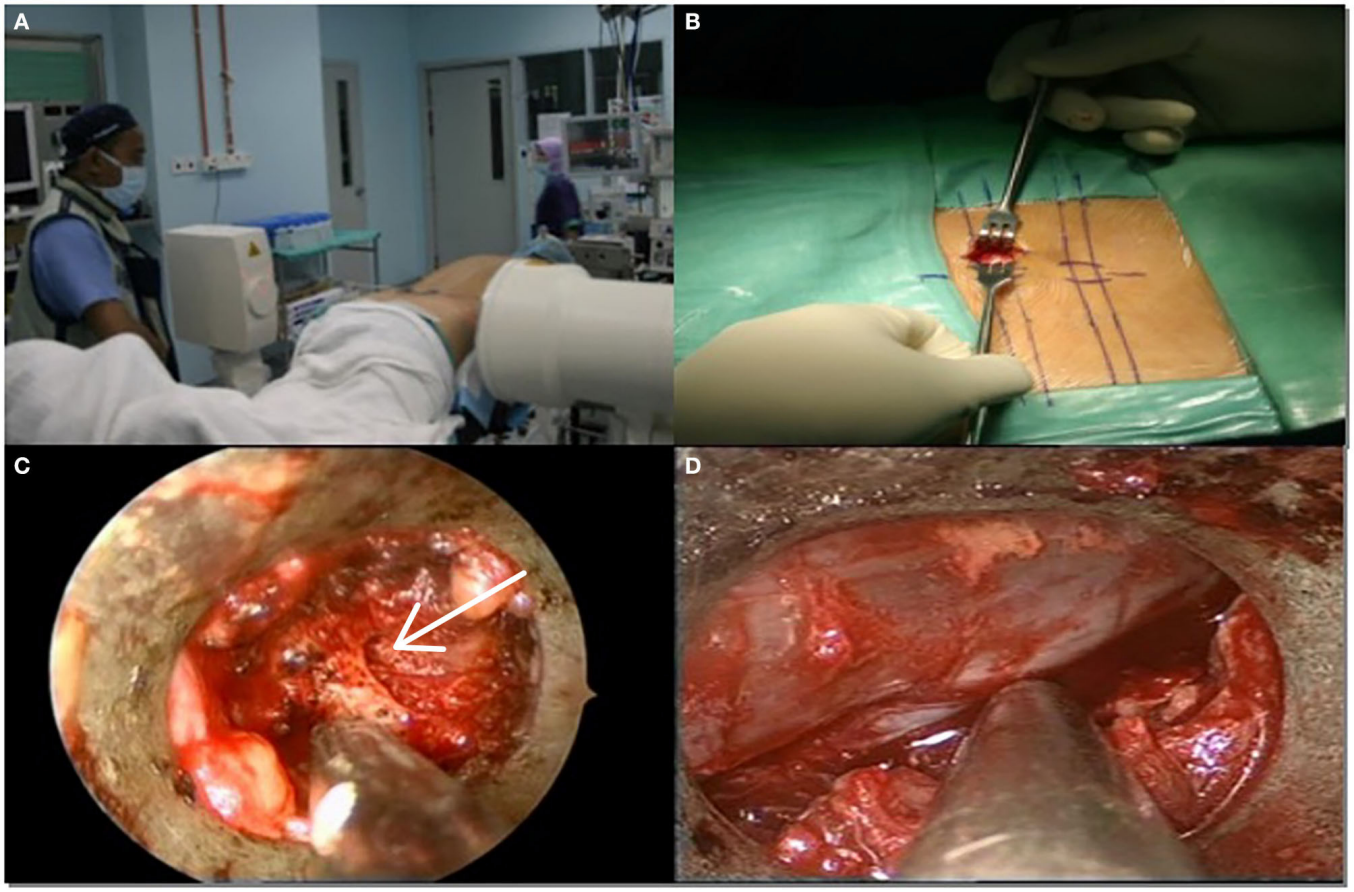
**(A)** Positioning and level marking. **(B)** Incision length was about 23 mm. **(C)** Spinolaminar junction was identified (white arrow). **(D)** Decompression was completed after traversing nerve root fully mobilized.

As for the surgery procedure, the patient underwent general anesthesia, and when the patient was fully anesthetized, the patient was brought into a prone position on the Jackson table (Figure 1). The patient's knees and hips were flexed at 40° and 45°, respectively, to increase the space of the interlaminar window. The abdomen was left free to avoid an increase in intra-abdominal pressure, which could cause an increase in bleeding capacity due to venous pooling during the operation. All body prominences were padded with soft silicon, and fluoroscopy was used to localize the vertebral level of the surgery.

The surgery began with a 23-mm craniocaudal incision made through the skin and muscle fascia, and the paravertebral muscles were retracted laterally to expose the lamina. Then, the endoscopic portal was inserted with its beveled opening toward the spinous process to place the 6.9-mm-outer-diameter rod-lens optic camera (Karl Storz Destandau endospine system). The rod-lens optic has a 9-mm diameter of an intraendoscopic exocentric-working canal with an angle vision of 0°. An intermittent normal saline flush was used to facilitate the maneuverability and clear the surgical field.

The next step involved the hemilaminotomy for the bilateral decompression approach in which the ipsilateral decompression was done by laminotomy of the inferior border of cranial lamina and flavectomy. Then, a high-speed burr was used to open the inferior border of the superior lamina to conduct the contralateral decompression by using the sublaminar approach.

The decompression was observed to be complete once the following outcomes were achieved. First, the traversing nerve root was freed and mobilized around 1 cm from lateral to medial. Second, there was no significant active bleeding within the spinal canal. Lastly, there were no free disk fragments visualized within the disk space or spinal canal. The instruments were then removed, and local steroid was given. Finally, watertight closure for lumbar fascia and subcutaneous tissue was done, and no drain was needed.

## Results

The mean follow-up period was 30.1 months (range = 17.2–43 months). There were 23 (38.3%) males and 37 (61.7%) females (Table 1) with a mean age of 60.82 years. Fifty-three patients had underlying medical comorbidities, which were diabetes mellitus in 18 patients (30%), hypertension in 32 patients (53.3%), and obesity in three patients (5%). Six patients (10%) had a mild degree of degenerative lumbar scoliosis, of whom two had a 12° angle, one had a 15° angle, two had a 17° angle, and one had a 22° angle. Forty-one (68.3%) of the patients had chief complaints of both back and leg pain (pain equal on both parts), whereas 5 (8.3%) complained of back pain (back pain more than leg pain), and 14 (23.3%) complained of leg pain (leg pain more than back pain).

**Table 1:** Biodemographic data of patients who underwent endoscopic lumbar stenosis surgery.

**Variables**	**Mean (SD)**	**Frequency (%)**
**AGE (YEARS)**		
40–49		7 (11.7)
50–59		15 (25.0)
>60		38 (63.3)
**GENDER**		
Male		23 (38.3)
Female		37 (61.7)
**Comorbid**		
Diabetes mellitus		18 (30.0)
Hypertension		32 (53.3)
Obesity		3 (5.0)
**SCOLIOSIS**		
Yes		6 (10.0)
No		54 (90.0)
**SPONDYLOLISTHESIS**		
Grade 1		12 (11.7)
Grade 2 and above		3 (5.0)
**CHIEF COMPLAINT**		
Back pain		5 (8.3)
Leg pain		14 (23.3)
Both		41 (68.3)
Operation time (h)	3.06 (0.98)	
Blood loss (ml)	150.18 (119.36)	
Post-operative hospital stay (days)	2.45 (1.11)	
**OPERATION LEVEL INVOLVE**		
L1–L2		0 (0.0)
L2–L3		3 (3.1)
L3–L4		19 (19.6)
L4–L5		51 (52.6)
L5–S1		24 (24.7)
Single-level surgery		28 (46.7)
Two-level surgery		26 (43.3)
Three-level surgery		6 (10)
Follow-up (month)	30.10 (12.98)	

The mean operation time was 183.6 min (ranging from 124.8 to 242.4 min). Mean blood loss was 150.18 mL (ranging from 30.82 to 269.54 mL). Post-operatively, mean hospital stay was 2.45 days (ranging from 1.34 to 3.56 days). A total of 97 levels were decompressed in 60 patients. The most frequently involved level was at L4/L5 in 51 patients (52.6%), followed by L3/L4 in 19 (19.6%), L5/S1 in 24 (24.7%), and L2/L3 in three (3.1%). Furthermore, 28 patients (46.7%) had single-level, 26 (43.3%) had two-level, and six (10%) had three-level decompression, all through a single surgery.

Paired *t*-test analysis revealed that there was a significant mean difference of VAS for back and leg pain between pre-operation and post-operation (*p* < 0.001; Table 2). There was no statistically significant difference in reduction in motor and sensory grading of the subjects, which was *p* < 0.673 and *p* < 0.784, respectively. The ODI showed a significant difference between pre– and post–endoscopic surgery (*p* < 0.001). For MacNab's classification, most of the endoscopic surgery outcomes were good in 28 (46.7%), excellent in 25 (41.7%), and fair in 7 (11.7%) patients (Table 3).

**Table 2:** Clinical outcome of pain using the visual analog scale (VAS), Oswestry Disability Index (ODI), and motor and sensory function pre- and post-endoscopic surgery.

**Variable**	**Pre-operation Mean (SD)**	**Post-operation Mean (SD)**	**Mean difference (95% CI)**	***p*-value**
**VAS**				
Back pain	4.13 (3.03)	0.87 (0.99)	3.279 (2.60, 3.94)	<0.001^*^
Leg pain	5.22 (2.59)	1.18 (1.24)	4.03 (3.37, 4.69)	<0.001^*^
Oswestry Disability Index	59.63 (20.66)	16.93 (12.02)	42.70 (36.86, 48.54)	<0.001^*^
MRC grading for leg strength	4.87 (0.38)	4.83 (0.59)	0.03 (−0.124,0.19)	0.673
ASIA scoring for sensation	1.65 (0.55)	0.87 (0.99)	−0.02 (−0.14, 0.11)	0.784

* *Statistically significant*.

**Table 3:** Clinical outcome of surgery based on MacNab's criteria.

**Classification**	**Frequency (%)**
Excellent	25 (41.7)
Good	28 (46.7)
Fair	7 (11.7)
Poor	–

## Discussion

Interlaminar endoscopic spinal stenosis surgery is currently becoming well-known because of its paramount advantages as it does not contribute to massive scarring of epidural space, which normally occurs in conventional laminectomy that may lead to the tethering of the cauda equina nerve roots (11, 12). This procedure also spares the soft tissue and bone of the spine because of its minimal resection, thus preserving the stability of the spine (13–15). The advantage of its mini–open surgery is becoming popularized especially in the discectomy procedure (16, 17).

However, no study compared the long-term outcomes of endoscopic lumbar stenosis surgery with conventional decompressive surgery for lumbar stenosis in the literature. The only similar studies are those on endoscopic discectomy. According to a study by Ruetten et al. (18), by using interlaminar endoscopic surgery, 89% of patients would undergo similar operation because of its good outcome, and additionally, 90% of patients were relieved of sciatica and satisfied with the procedure (19–21). This result is comparable with that of a prospective and randomized study of surgical treatment for lumbar disk herniation by Hermantin et al. (22), in which the satisfactory result was obtained in 97% in the endoscopic group (*n* = 30) and 93% in open laminectomy group (*n* = 30).

In another prospective, randomized, and controlled study of 178 patients who underwent full endoscopic surgery, 97% (*n* = 88) in the endoscopic group achieved satisfaction compared with 88% (*n* = 77) in the conventional microsurgical group (23). The author subdivided the endoscopic group into the interlaminar and transforaminal techniques. In contrast to the interlaminar technique, the transforaminal technique can be conducted easily and has better visualization into canal structure but has an inoperable sequestrated disk and a very limited exposure to ligamentum flavum. Additionally, because of the anatomical hindrance at the L5–S1, the interlaminar technique can tackle the challenge of adequate decompression. The exposure of yellow ligament was also widely visualized, which enabled the surgeon to remove thickened flavum in degenerative stenosis.

As for the present study, a total of 60 patients were eligible during the decided period: January 2009 to December 2013. The mean age of the patients was 60.82 years, and the mean follow-up period was 30.1 months. The mean operation time was 183.6 min (ranging from 124.8 to 242.4 min). The present study is similar to a prospective study by Khoo and Fessler in which the mean operation time was 109 min per level (ranging from 45 to 240 min) (24). The operative time was longer during the initial learning curve, which involved approximately 30 cases. Unlike a prospective study by Pao et al., who found a mean of 126.7 ± 38.3 min for single-level decompression (25), we devoted approximately 2 h for a single-level decompression, that is, the “skin-to-skin” process. We were also contemplating on the best position for the patient on the table and the best view for skin marking. However, we managed to reduce the operative time for decompression by using a high-speed burr.

The mean blood loss was 150.18 mL (ranging from 30.82 to 269.54 mL). Khoo et al. and Pao et al. reported a blood loss of 68 mL (ranging from 15 to 300 mL) and 104.5 mL (ranging from 21.7 to 230.7 mL) (24, 25). In a study by Kaushal et al., the mean blood loss was 45 mL (ranging from 30 to 70 mL) with an operative time of 50 min (ranging from 40 to 80 min) in interlaminar endoscopy discectomy (19). Martin et al. and Komp et al. reported nil blood loss intraoperatively (1, 18). This is due to the endoscopic system that they used (WOLF system), which allowed continuous lavage and possibility of radiofrequency, bipolar preparation, and coagulation (1). Post-operatively for the present study, there was no suction drainage and blood transfusion required, and the mean hospital stay was 2.45 days (ranging from 1.34 to 3.56 days). This duration was comparable with those in the study by Komp et al., Chen et al., and Choi et al., which was 3, 1.4, and 3.83 days, respectively (1, 26, 27).

The overall results of the present study show a statistically significant reduction of pain for both back and leg, which concurs to various literature (1, 18, 26, 27). Ryu et al. reported that there was a significant reduction of VAS post-operatively in interlaminar endoscopic surgery (28). They found that the mean VAS for back and leg pain decreased from 5.2 to 2.4 and 7.6 to 1.8, respectively. Their mean follow-up was 26 months. Another study that shared similar positive results was by Lee et al. (29), who showed a statistically significant reduction of VAS for back and leg pain of 2.8–2.3 and 7.4–2.1 (*p* < 0.05), respectively. The results of the previous studies are similar to those of the present study, in which the VAS for back and leg pain decreased from 4.13 to 0.87 and 5.22 to 1.18 (*p* < 0.001), respectively, with a total mean duration follow-up of 30.1 months.

However, there was a reduction of muscle power and sensory grading after the endoscopic decompression. Statistically, the reduction was insignificant to the present study. The reason might be due to the long-standing degenerative compression to the nerve root. Unfortunately, no previous studies compared the post-operative neurological outcomes.

As for the clinical outcomes, the parameter that was used in the present study was the ODI, which showed a significant improvement in the patients' daily activities (*p* < 0.001). A prospective study by Komp et al. (1) showed a constant and significant reduction in leg pain and improvement in daily activities (*p* < 0.001). The same findings were found in Ryu and Seocho-gu (28) and Ruetten et al. (30), which showed similar good clinical outcomes regarding the ODI.

For clinical outcomes based on MacNab's criteria, the present study showed a good outcome and was comparable with other endoscopic studies. Chang et al. described MacNab's criteria as 85% for both excellent and good outcomes and 8% for each fair and poor outcome (31). Another study that showed similar results was by Lee et al. (29), in which 92.1% of patients had excellent to good outcomes and 7.9% of patients had fair to poor outcomes. For the present study, 88.4% showed excellent to good outcomes, 11.7% showed fair outcomes, and none of the patients showed poor outcomes. There were two reasons for the fair outcomes, namely, (1) three patients were found to have a lot of fibrosis intraoperatively, which had caused some difficulties in dissection; and (2) four patients had significant facetal arthrosis besides severe claudication from stenosis. Because they refused fusion, we opted for decompression surgery for them. Long-term follow-up for open decompressive laminotomy for degenerative LSS showed a lower percentage outcome: 56.7% of excellent to good improvement (32).

As for complications, incidental durotomies were inevitable. This complication can be minimized by carefully separating the yellow ligament from the dura and placing a small patty in between before excision of the thickened ligament. The use of 90° Kerrison punch instead of 45° to excise the ligament would be helpful in this context. The ligament is not cut unless dura is clearly seen, and Kerrison punch is used with caution when entering the canal space to ensure that it bites the bone and not the soft tissue.

Another complication that is considered essential is marking the wrong level. This is the initial learning curve, in which the technique of marking the level is done in a lateral view. Subsequently, the marking of level on the skin is done in an anteroposterior view, and further line markers are used to ensure 100% accuracy. The presence of transitional vertebrae must be ascertained to avoid wrong numbering. Additionally, inadequate decompression would happen during the learning curve if the surgeon is too cautious in removing the bone and the soft tissue. This is because handling proximal levels such as L2/L3 and L3/L4 is a great challenge. It should be approached slightly differently because space is tighter and has a higher risk of nerve root injury. Therefore, dissection is done more extensively before reaching the stenotic area. The retraction of nerve root must be done cautiously to avoid unnecessary exploration.

Pertaining to the unilateral hemilaminotomy and bilateral decompression, Oertel et al. demonstrated an 85.3% excellent to fair operative result, which was comparable with our result (33). The same improvement of pain with adequate preservation of vertebral stability was demonstrated by Halit et al., where ODI scores decreased significantly in both early and late follow-up (34). Several limitations were observed in the present study, among which was the small sample size that did not represent most ethnicities in Malaysia and the difficulty to follow up the patients because of several reasons, such as invalid contact numbers of the patients. A study to evaluate the predictive factors of endoscopic surgery for lumbar stenosis can be done in the future to analyze the association between medical factors and the outcome of the surgery.

## Conclusion

To summarize, unilateral percutaneous endoscopic spine surgery for the bilateral decompression in lumbar stenosis provides excellent outcomes, yet safe and effective operation. Despite some limitations and few complications, higher success can be achieved if certain precautions are taken. Significantly, the surgery improves back and leg pain and the patient's function.

## Data Availability Statement

The raw data supporting the conclusions of this article will be made available by the authors, without undue reservation.

## Ethics Statement

The studies involving human participants were reviewed and approved by Human Research Ethics Committee, Universiti Sains Malaysia. The patients/participants provided their written informed consent to participate in this study.

## Author Contributions

AS and AY: study design and reviewer. JJ and MY: reviewer. All authors contributed to the article and approved the submitted version.

## Conflict of Interest

The authors declare that the research was conducted in the absence of any commercial or financial relationships that could be construed as a potential conflict of interest.
